# Ocular dominance plasticity: inhibitory interactions and contrast equivalence

**DOI:** 10.1038/srep39913

**Published:** 2017-01-10

**Authors:** Daniel P. Spiegel, Alex S. Baldwin, Robert F. Hess

**Affiliations:** 1 McGill Vision Research Department of Ophthalmology McGill University, Montreal, Canada

## Abstract

Brief monocular occlusion results in a transient change in ocular dominance, such that the previously patched eye makes a stronger contribution to the binocular percept after occlusion. The previously unpatched eye therefore makes a correspondingly weaker contribution to the binocular sum. To shed light on the mechanism underlying this change we investigate how the relationship between the perception of fusion, suppression, and diplopia changes after short-term monocular deprivation. Results show that fusible stimuli seen by the unpatched eye are actively suppressed as a result of patching and that this can be reversed by an interocular contrast imbalance. This suggests that dichoptic inhibition plays an important role in ocular dominance changes due to short-term occlusion, possibly by altering the contrast gain prior to binocular summation. This may help explain why this form of plasticity affects the perception of both fusible and rivalrous stimuli.

Monocular occlusion has been a popular model for studying visuocortical plasticity for a long time. Since the time of the pioneering work of Weisel and Hubel^1^,^2^,^3^ (for a short overview see^4^) there have been numerous reports on the effects of monocular occlusion both in animal and humans. It is well-described that prolonged periods of monocular deprivation have marked effects on ocular dominance^5^,^6^ and interestingly, brief periods of monocular occlusion are more powerful than the same amount of time administered in one or two periods^7^,^8^.

Similar observations have been made in human experiments. Tyler *et al*.^9^, showed that monocular deprivation from high spatial frequencies increased visual evoked potentials (VEP) in the non-deprived eye during the period of deprivation and reduced the response of the deprived eye. On the other hand, Zubek *et al*.^10^, showed changes in critical flicker frequency (initially a decrease followed by an increase) in the non-occluded eye over the course of 24-hour period of visual deprivation whereas the deprived eye showed no change. Lou *et al*.^11^, showed that a period of 24 hours of monocular occlusion resulted in a decrease in cortical excitability. In general, these early studies showed that a monocular deprivation has a measurable effect on the binocular system by reducing sensitivity of the deprived eye and possibly enhancing sensitivity of the non-deprived eye.

More recent studies in human observers revealed an intriguing phenomenon where after a short period (1–3 hours) of monocular deprivation, the ocular dominance plasticity is characterized by a strengthening of the input from the previously patched eye and a weakening of the input from the unpatched eye. The imbalance is reflected in the change in binocular rivalry^12^ and in the contribution that each eye makes to a fused binocular percept^13^ both at^13^ and above threshold^12^,^13^. Two very different approaches have been used to document these changes: binocular rivalry and measurement of fusional balance using phase-shifted sinusoids. That these two different approaches both suggest similar neuroplastic changes gives a clue to the underlying neural mechanism. A unified explanation could be advanced in terms of the current two-stage model of binocular combination^14^ where there are inhibitory inputs to the monocular contrast gain controls prior to excitatory combination (Fig. 1). Although these interactions occur before binocular combination they would still result in altered rates of rivalry for non-fusible stimuli as well as a change in dominance for fusible stimuli for example manifested as a shift of the position of the fused image in the direction of the dominant eye. Further because their influence is on the monocular contrast gain, these changes may be equivalent to a simple change in interocular contrast.

In this study, we adopted an elegant task recently introduced by Georgeson and Vallis^15^. Using a simple visual stimulus, blurred horizontal edge with different levels of blur/spatial scale (1, 2, 4, 8, 16, and 32 arcmin), vertical disparity (0, 1, 2, 3, 4, 5, 6, 7, and 8-times blur width), and interocular contrast offset (0, 6, and 12 dB) allowed them to evaluate the inter-relation between fusion, suppression, and diplopia in normal and abnormal visual conditions using one unified model. Among other, Georgeson and Wallis^15^ have shown that in normal binocular vision fusion dominates at small vertical disparities and diplopia (which they explained as a lack of both fusion and interocular suppression) at large vertical disparities corresponding to the stimulus scale. Suppression of one eye by the other, as a mechanisms maintaining a single vision outside of fusional disparities was less prevalent, and peaked at intermediate disparities. Their measurements revealed that the fusional range was roughly scale invariant, but that the range of suppression was greater at finer scales than would be predicted under scale invariance. To date the ocular dominance changes that occur from short-term monocular occlusion have been demonstrated using medium spatial frequency stimuli that cannot be fused (i.e., binocular rivalry^16^,^17^) or low spatial frequency stimuli that are fused (i.e., phase combination^13^, motion combination^18^, and contrast combination^19^). A common explanation in terms of the suppressive interactions between the two eyes prior to binocular combination could explain the findings for both rivalrous and fusible stimuli across the spatial scales used in previous studies.

In this study, we aim to explore whether it is the inhibitory interactions that lie at the heart of the patching-induced changes in dominance. We reason that if they occur in a scale-independent but contrast-dependent fashion we can advance a unified explanation in terms of a change in contrast gain arising from imbalanced inhibitory contralateral signals at a relatively early stage of visual processing prior to binocular combination. To investigate the relationship between patching and interocular contrast imbalance and their effect on binocular balance, we used a modified approach of Georgeson and Vallis^15^. The adaptation of their task was that we introduced a small tilt to the blurred edges with an opposite direction in each eye that allowed for a dissociation between fusion and suppression at small disparities. The edges varied in their vertical disparity and in their spatial scale. Data were collected before and after 3 hours of monocular translucent occlusion. The results suggest that short-term monocular deprivation increases the suppression of the unpatched eye at small vertical disparities where fusion usually dominates, in a scale invariant manner. We also found that we could reverse the induced patching effect using a relative increase of the stimulus contrast viewed by the unpatched eye.

## Materials and Methods

### Participants

Four participants with normal or corrected-to-normal vision (mean age 30 ± 4.3 SD, 1 female) with normal stereoacuity as determined by Randot stereotest took part in the study. Their biographical and visual characteristics are presented in Table 1. All participants were experienced psychophysical observers, and one of them was not naïve to the purpose of the experiment (first author). They gave informed consent before the first session. All procedures were conducted in accordance with the Code of Ethics of the World Medical Association (Declaration of Helsinki) and were approved by the Research Ethics Board of the McGill University Health Center.

### Stimuli

The stimuli were a pair of dichoptically-presented tilted edges (Fig. 2A) calculated as Gaussian integrals with a blur B = 4 or 32 corresponding to the sigma of the Gaussian (we will refer to it throughout as scale: 4 arcmin being the fine scale, 32 arcmin being the coarse scale) and a tilt of 2.36 degrees in opposite directions. Our edges are tilted to allow separation between fusion and suppression at small disparities. Stimulus disparity (in multiples of the corresponding scale, i.e. 0–8 × B) was varied across the trials while the stimulus contrast (offset of 0, 3, 6 or 12 dB) and stimulus scale (B = 4 or 32) was varied across blocks. In case of 0 dB contrast offset, both edges had 30% Michelson contrast. In case of 3, 6, and 12 dB contrast offset, the ratio between the contrasts presented to the two eyes was a factor of 1.4, 2, and 4 respectively, resulting in contrasts of 35.5% and 25.4%, 42.4% and 21.2%, and 60% and 15% presented to the two eyes. The edges were enclosed within a binary noise frame to control vergence and were accompanied by two black reference horizontal lines. The whole stimulus excluding the reference lines was scaled to 2.1 and 16.8 degrees for the fine and coarse edges, respectively. Polarity of the edges, i.e. darker top or bottom, was randomly chosen on each trial with both edges having the same polarity. The tilt directions were also randomly chosen on each trial and were always in opposite directions in the two eyes.

### Procedure

Participants viewed a gamma-corrected ViewSonic V3D231 white (x = 0.305; y = 0.315 z = 0.380) 3D LED display (refresh rate = 60 Hz; mean luminance through polarizers = 25.6 cd/m^2^) with polarized glasses (worn over their corrective lenses if necessary- crosstalk = 1%) from a distance of 72 cm. The display was driven by an iMac 11.1 (MacOSX, Intel Core i7, 2.8 GHz, 8 GB RAM). Stimuli were created using Psychtoolbox^20^,^21^ for MATLAB (MathWorks, Natick, MA). On each trial the stimulus was presented for 200 ms, accompanied by a simultaneous auditory signal (100 ms, 880 Hz click). A single-interval, 4-alternative choice design was used. The task was to report the binocular percept using a keyboard. The responses were: single flat edge (no tilt), single right-tilted edge (i.e. right side higher), a single left-tilted edge (i.e. left side higher), or two (tilted) edges. These reflected fusion, suppression of either eye, or diplopia, respectively. Each response initiated the next trial after a 300 ms interstimulus interval.

The whole experiment consisted of two blocks of eight experimental conditions (4 contrast offsets × 2 scales), each containing 144 trials, performed before and after 3 hours of patching of the non-dominant eye. The eye dominance was established by the the Miles test^22^. The total number of trials was therefore 2304. Before each block, participants aligned the percepts of the two eyes using a dichoptically presented nonius cross. Every participant was provided with at least 144 training trials to familiarize him or her with the task prior to any data collection.

### Data analysis

We converted responses into proportions of “Single flat edge”, “Single tilted edge”, and “Two edges” as a function of vertical disparity. The “Single tilted edge” responses were split between those corresponding to the tilt of the edge shown to the non-dominant and the dominant eye. We calculated proportions for the three different conditions: 0, 3, and 6 dB contrast offset favouring the patched (PE) or unpatched eye (UPE) both pre- and post-patching. The data were then averaged across the participants.

To quantify the relationship between fusion, suppression, and diplopia, we employed a modified version of the model presented in Georgeson and Wallis^15^ (Fig. 3). The decision tree is depicted in Fig. 3. When the two edges are fused we expect that the percept is a single flat edge (i.e. no tilt). Fusion is possible only over a limited range of disparities. Our model presumes, similarly to the one of Georgeson & Wallis^15^ that the highest probability of fusion occurs at zero disparity (*pFuse*_*0*_) and it falls with increasing disparity as a generalized Gaussian function:


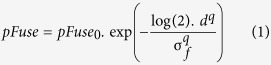


where d = disparity, σ_f_ = fusional range, and q = fusion steepness. We also follow their work^15^ in assuming that monocular percepts are eliminated when fusion occurs.

If fusion does not occur, the visual system is in the non-fused state and the next available mechanism that allows for single vision is suppression. We expect that the visual percept resulting from suppression is a single edge with a tilt corresponding to the one of the unsuppressed eye (*tiltSupp*). Following Georgeson and Wallis^15^ we suppose that the probability of suppression is maximal at zero disparity (*pSupp*_*0*_) and it falls as a Gaussian function with increasing disparity:


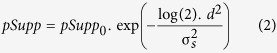


d = disparity and σ_s_ = suppression range. Suppression results a monocular state whose probability (*pMonoc*) can be expressed as:


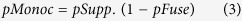


The probability that the patched eye’s percept is preserved in the monocular state is calculated as:


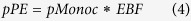


where EBF is the Eye Balance Factor ranging between 0 and 1; 1 = full dominance of the Patched eye; 0 = full dominance of the Unpatched eye. The probability that the percept of the unpatched eye is preserved is modelled as


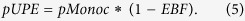


Finally, the probability of diplopia was calculated as


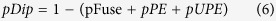


Modelled responses are depicted as solid lines through the behavioural data in Fig. 4. Following Georgeson and Wallis^15^ we fixed the fusion steepness at *q* = 4 to reduce the number of free parameters. Our model then had four free parameters (Table 2) characterizing: 1. the probability of fusion at zero disparity (*pFuse*_*0*_), 2. the range of fusion (σ_f_), 3. the range of suppression (σ_f_), and finally 4. the balance between the eyes (*EBF*).

All data fitting was done using the nlinfit MATLAB routine. The goodness of fit for each condition was quantified by Equation 7:


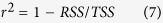


where


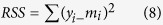


and


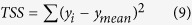


where *y*_*i*_ and *y*_*mean*_ refers to the data and *m*_*i*_ refers to the model prediction.

In addition to fitting the averaged data, we also fitted each participant’s data to derive the *EBF* parameter for the 0 and 3 dB conditions at both stimulus scales Pre and Post patching. The Pre and Post data were subjected to the paired t-test (Fig. 5). The same analysis was conducted for the pFuse0 parameter (see Supplementary information).

## Results

In Fig. 4A,B we show the normal pattern of binocular interactions for the two stimulus scales prior to 3 hours of monocular patching. In each case, the probabilities of responses are plotted along with solid lines showing the model fits. These are plotted as a function of vertical disparity in units of stimulus scale. For both scales fusion is dominant at low disparities (*pFuse*_*0*_ ~0.7), diplopia is dominant at large disparities, and suppression is absent except for a slight peak at intermediate disparities. After 3 hours of monocular patching of the non-dominant eye, the responses measured immediately after removal of the patch are shown in Fig. 4C,D. The main effect of the patching is to increase the inhibition of the previously unpatched eye (red triangles). This increase is seen at both stimulus scales and is particularly evident at low to medium disparities. This shift in dominance is also indicated by a shift in the fitted *Eye Balance* parameter from 0.41 and 0.62 to 0.69 and 0.91 at fine and coarse scale, respectively.

To determine whether the patching effect was equivalent to a change in input contrast gain we assessed whether its effects could be reversed by an interocular contrast offset. Results displayed in Fig. 4E,F show the pre-patching effect where the stimulus contrast viewed by the patched eye is reduced by 3 dB relative to the stimulus contrast viewed by the unpatched eye. The effect of the contrast imbalance is to counterbalance the improved sensitivity of the patched eye. In other words, increasing the relative contrast to the unpatched eye negates the effects of patching. Results displayed in Fig. 4G,H show the post-patching effect where the stimulus seen by the patched eye is reduced in contrast by 3 dB relative to that of the unpatched eye. Patching acts to reverse the pattern of dominance produced by the contrast imbalance but does not achieve pre-patching levels (Fig. 4A,B), i.e. there is greater inhibition of the unpatched eye as reflected by the increase of the *Eye Balance* parameter from ~0 to ~0.3 for both stimulus scales (Fig. 5). This shows that the effect of a 3 dB offset is slightly stronger than needed to neutralize the patching effect. This trend is exaggerated for larger contrast offsets (data for 6 and 12 dB not shown).

The relative effects of patching and contrast offset are summarized in Fig. 5 where the *Eye Balance* parameters derived from the model fits are compared for the various conditions.

To evaluate how contrast offset affects the dominance change that results from patching we assessed the fitted *Eye Balance Factor* parameter (provided in Table 2). For both the course and fine scales and at both contrast ratios patching increased the value of the fitted EBF by approximately 0.3. For the fine scale condition the EBF for the 3 dB offset after patching was similar to that for a balanced contrast before patching (within the parameter’s error that we derived from the fitting). This indicates that the patching effect on eye balance can be counteracted by a change in physical contrast. For the coarse scale condition the post-patching value with the 3 dB offset was lower than the pre-patching value with balanced contrast – indicating that this physical contrast offset had a stronger effect than the patching change.

To provide further statistical support, we conducted a repeated measures analysis of variance (ANOVA) with within-participant factors of time (Pre and Post patching), contrast offset (0 dB and 3 dB favoring UPE), and scale (Fine and Coarse) followed by a paired t-test. This was done after first determining that the data were normally distributed using the Shapiro-Wilk test (p > 0.23). The ANOVA revealed the main effect of time (F_1, 3_ = 68.83, p = 0.004, η^2^ = 0.96) as well as a significant effect of contrast offset (F_1, 3_ = 37.76, p = 0.001, η^2^ = 0.93). No interactions reached statistical significance. Patching shifted eye dominance towards the patched eye irrespective of stimulus scale (purple vs. yellow bars in Fig. 5). Applying a contrast offset before patching results in a significant reversal of this effect (fine scale t_3_ = 6.06; p = 0.009, coarse scale t_3_ = 8.2; p = 0.004). When this offset is applied after patching it negates the dominance change that is produced as a result of short-term patching. As a result, the difference between the pre-patching dominance (pre-purple) and the 3 dB post patching dominance (post-yellow) was no longer significant (fine scale t_3_ = 1.36; p = 0.25, coarse scale t_3_ = 1.3; p = 0.29). For analysis of the pFuse0 parameter see Supplementary information.

## Discussion

By examining how the relationship between suppression, fusion and diplopia changes as a result of short-term monocular deprivation we sought to address two questions; first is the effect of short-term monocular deprivation to imbalance the binocular inhibitory interactions for fusible stimuli ?, second, are the patching-induced changes independent of spatial scale but contrast dependent? The first question is relevant to the previous approaches that have been used to quantify the effects of short-term plasticity due to monocular occlusion. Binocular rivalry reflects inhibitory modulation between stimuli that cannot be fused^12^ whereas quantifying the contribution of each eye to the binocularly fused percept^13^ is relevant only for fusible stimuli. We sought to answer whether the effects seen in these two approaches occur in the same underlying mechanism as would be the case if the patching effects targeted the contralateral inhibitory stage prior to binocular summation^14^. To answer this we need to know if short-term patching modulates interocular inhibition *for stimuli that can be fused* as we know it does for stimuli that cannot be fused^14^. The results show that there is a change in the inhibitory interaction for fusible stimuli, suggesting that the site of ocular dominance plasticity may be at the level of the contralateral inhibitory interactions prior to binocular summation. This does not depend on stimulus spatial scale. We also show that this change in ocular dominance can be reversed by a compensatory change in the interocular contrast, consistent with the notion that this neuroplastic change involves a reciprocal adjustment to contrast gain in the two monocular pathways prior to binocular summation.

**Figure 1:**
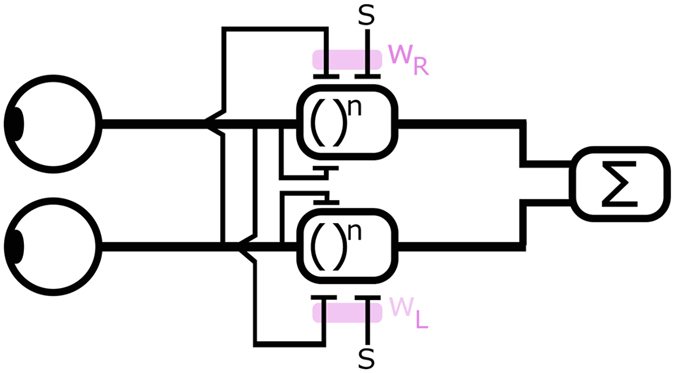
The first stage of a two stage model of binocular combination in which contralateral inhibitory interactions (W_R_ and W_L_) control the contrast gain of the first stage prior to binocular combination (adapted from^14^).

**Figure 2:**
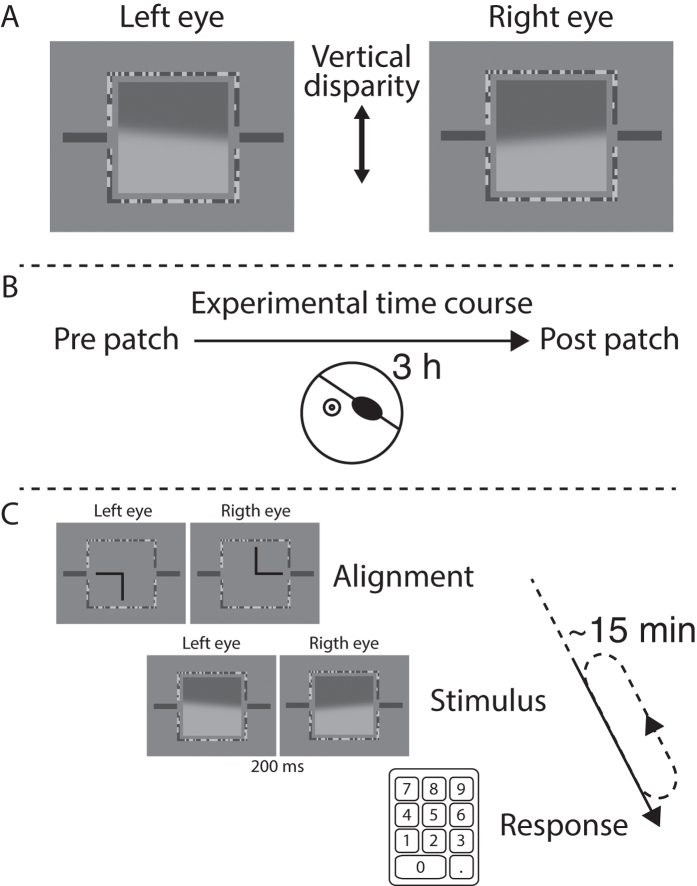
Top, Stimuli (Panel A) were dichoptically presented tilted blurred edges. Each edge was tilted by 2.36 degrees from the horizontal direction. The tilt was always in the opposite direction between the two eyes. Panel A depicts an example condition with 0 contrast offset, 0xB disparity, left tilt to the left eye, and right tilt to the right eye. Panel B shows the time course of one experimental session. Panel C shows the time course of one experimental block.

**Figure 3:**
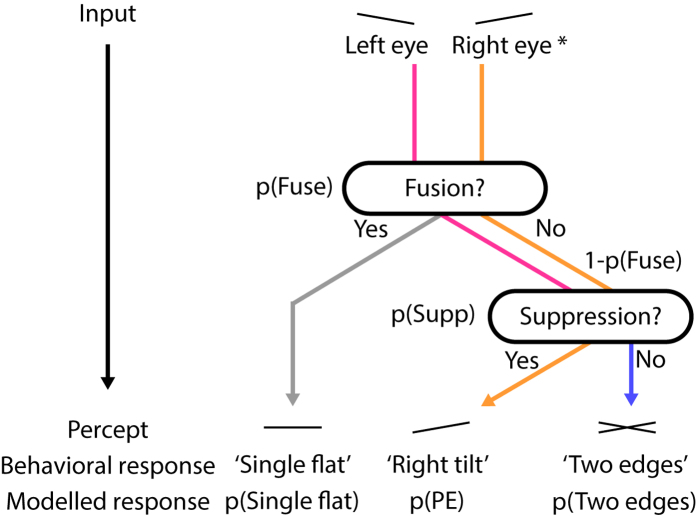
Relationship between the input, percept, behavioral responses, and modelled responses. In this example, right eye is the patched eye (PE; indicated by *) and is presented with a right tilted edge.

**Figure 4:**
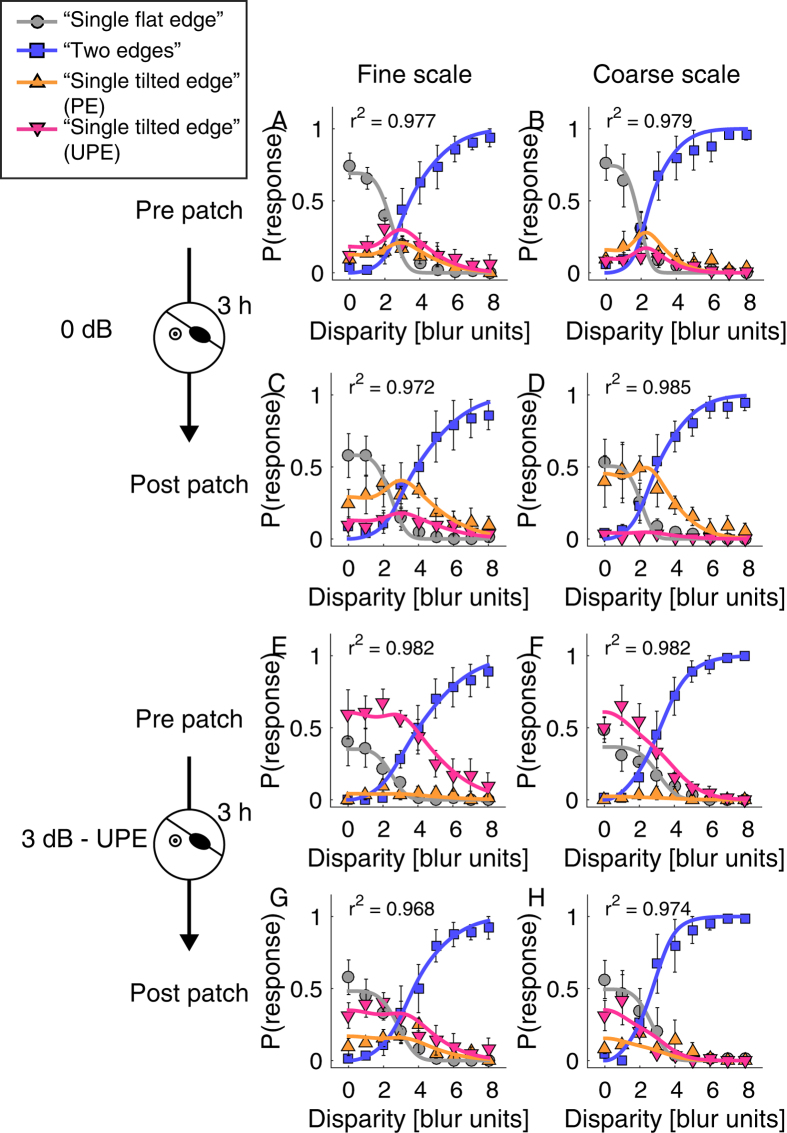
The effect of patching and contrast imbalance. Y axis of each plot shows the probability of occurrence of each response as a function of interocular vertical disparity in units relative to the degree of scale. Top row (panels A,B) shows 0 dB contrast offset pre patching; second row (panels C,D) shows 0 dB contrast offset post 3 hours of patching. Panels E and F show 3 dB favoring the unpatched eye (UPE) pre patching and panels G and H show 3 dB favoring the unpatched eye (UPE) post patching. Error bars ± SE. The solid lines are model fits with r^2^ reported in each plot.

**Figure 5:**
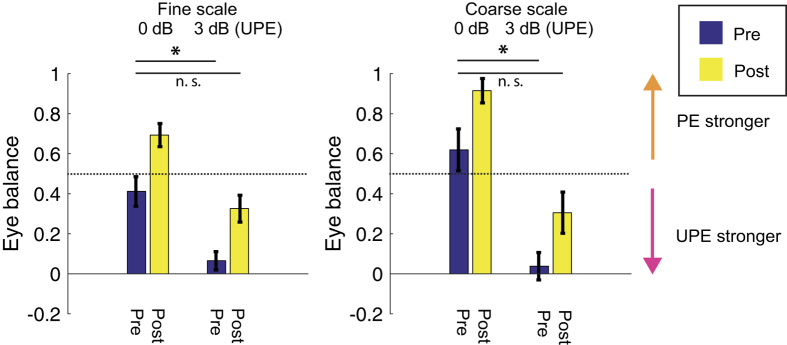
The effect of patching on the fitted Eye Balance Factor (EBF). EBF ranges between 0 and 1; 1 ~full dominance of the patched eye, 0 ~full-dominance of the unpatched eye. Each pair of bars show the fitted value pre- (Purple) and post-patching (Yellow). The left pair in each panel shows the 0 dB contrast offset, right pair shows the 3 dB offset favoring the unpatched eye. The dotted line shows balanced input from the two eyes. Error bars depict the standard error of the parameter determined by the fitting routine. *p < 0.05 for a paired t-test.

At first glance it is surprising that two approaches that are so different provide such congruent estimates of how ocular dominance is altered by short-term monocular occlusion. Although binocular rivalry involves exclusively non-fusible stimuli and hence cannot reveal anything about binocular combination, there is a view that rivalry and dichoptic masking engage the same (inhibitory) neural mechanisms in the visual pathway^17^,^23^. In which case, as a consequence, binocular rivalry may reflect the dichoptic inhibitory interactions (illustrated in Fig. 1) that are modulated by short-term monocular occlusion and which ultimately limit the extent of binocular fusion for stimuli viewed by the left and right eyes. We propose that these changes occur through contrast gain control. Support for this assumption comes from recent data from intrinsic optical imaging in non-human primates indicating that the effects of short-term monocular deprivation have their origin in corresponding cellular changes at the level of ocular dominance columns in V1^24^.

In our study, we did not intend to measure the time course of the induced perceptual changes. We assume that the changes are only short-lasting as previous studies showed that the effects of short-term monocular occlusion last about 30 minutes for fusible^13^ and 90 minutes for non-fusible stimuli^12^.

It has been also shown that the effects of monocular deprivation are tightly associated with GABAergic inhibition within the visual cortex^25^. Considering that the effects of patching were largely associated with increased suppression of the previously unpatched eye, we speculate that a neural mechanism related to GABA-mediated inhibition is the culprit of the observed changes. If this is the case non-invasive brain stimulation techniques such as transcranial magnetic or direct current stimulation, known to alter intracortical inhibition^26^,^27^,^28^ may influence the effects in terms of duration and/or magnitude.

Our results also have potential clinical implications in visual disorders characterized by binocular imbalance such as amblyopia. Indeed, it has already been shown that patching of the amblyopic eye strengthen its contribution to the binocular visual system^29^. Here, we contribute to the suggestion that inverse occlusion (i.e. occlusion of the amblyopic eye) has merit as an alternative clinical intervention alone or in combination with traditional^30^,^31^,^32^ or with emerging treatment regimens^33^,^34^,^35^,^36^,^37^.

## Additional Information

**How to cite this article**: Spiegel, D. P. *et al*. Ocular dominance plasticity: inhibitory interactions and contrast equivalence. *Sci. Rep.* **7**, 39913; doi: 10.1038/srep39913 (2017).

**Publisher's note:** Springer Nature remains neutral with regard to jurisdictional claims in published maps and institutional affiliations.

## Supplementary Material

Supplementary Information

## Figures and Tables

**Table 1:** Visual characteristics of the 4 normal observers tested.

ID	Gender/Age	Eye	VA [log MAR]	Stereo [arc sec]	Refraction [D]
P1	M/34	RE	−0.3	40	0
		LE	−0.3		0
P2	F/24	RE	0.0	40	−4.0
		LE	−0.02		−3.75
P3	M/32	RE	−0.2	40	−1.25 −2.75 × 180
		LE	−0.2		−1.25 −2.75 × 180
P4	M/30	RE	−0.18	40	0
		LE	−0.18		0

**Table 2:** Model estimates of free parameters and estimate error: *pFuse*_*0*_ = probability of fusion at zero disparity, *σ*_*f*_* = *Fusional range, *σ*_*s*_* = *Suppression range. 3 dB UPE = 3 dB contrast offset favoring the unpatched eye.

Stimuli contrast offset [dB]	Time point	Scale	σ_s_ [blur units]	σ_f_ [blur units]	Eye Balance Factor [EBF]	pFuse_0_
0 dB	Pre patch	Fine	3.35 ± 0.20	2.37 ± 0.19	0.41 ± 0.07	0.69 ± 0.05
		Coarse	2.42 ± 0.18	1.88 ± 0.16	0.62 ± 0.10	0.74 ± 0.06
	Post patch	Fine	3.88 ± 0.20	2.42 ± 0.22	0.69 ± 0.06	0.58 ± 0.05
		Coarse	2.93 ± 0.15	2.06 ± 0.20	0.91 ± 0.06	0.50 ± 0.05
3 dB UPE	Pre patch	Fine	4.02 ± 0.17	2.48 ± 0.31	0.07 ± 0.05	0.35 ± 0.04
		Coarse	2.79 ± 0.18	3.05 ± 0.38	0.04 ± 0.07	0.37 ± 0.05
	Post patch	Fine	3.55 ± 0.22	2.84 ± 0.33	0.33 ± 0.07	0.48 ± 0.05
		Coarse	2.18 ± 0.24	2.70 ± 0.33	0.31 ± 0.10	0.49 ± 0.06
