# Supplementary material for: Ocular dominance plasticity: inhibitory interactions and contrast equivalence
Source: Sci Rep. 2017 Jan 10;7:39913. doi: 10.1038/srep39913 (PMC5223198; doi:10.1038/srep39913)
Supplement: Supplementary Information [file srep39913-s1.doc]

**Ocular dominance plasticity: inhibitory interactions and contrast equivalence – SUPPLEMENTARY MATERIALS**

Daniel P. Spiegel *, Alex S. Baldwin, and Robert F. Hess

McGill Vision Research

Department of Ophthalmology

McGill University, Montreal, Canada

* Corresponding Author:

Daniel P. Spiegel

McGill Vision Research Unit, Dept. of Ophthalmology

L7-213 Montreal General Hospital, Montréal, Québec, Canada, H3G 1A4

Email: daniel.spieg@gmail.com

Phone: +1 65 9857 7028

## Supplementary analysis of pFuse0

We conducted an additional statistical analysis on the *pFuse0* parameter. Normality of the data was evaluated by the Shapiro-Wilk test (p > 0.17). Again, we employed the repeated measures analysis of variance (ANOVA) with the same within-participant factors as for the *EBF* analysis, i.e. time (Pre and Post patching), contrast offset (0 dB and 3 dB favoring UPE), and scale (Fine and Coarse). Following the ANOVA, we used a paired t-test. The ANOVA revealed a significant effect of contrast (F_1, 3_ = 85.15, p = 0.003, η^2^ = 0.97). No other factors or interactions reached statistical significance. Imbalancing contrast between the two eyes significantly decreased the probability of fusion at zero disparity (fine scale; t_3_ = 3.33, p = 0.04, coarse scale, t_3_ = 10.44, p = 0.002) but patching did not reverse this decrease (fine scale; t_3_ = 6.84, p = 0.006, coarse scale, 4.72, p = 0.002) possibly due to the prevailing dominance (albeit non-significant; see the main text) of the unpatched eye induced by the contrast offset.
